# Redox-Sensitive Induction of Src/PI3-kinase/Akt and MAPKs Pathways Activate eNOS in Response to EPA:DHA 6:1

**DOI:** 10.1371/journal.pone.0105102

**Published:** 2014-08-18

**Authors:** Faraj Zgheel, Mahmoud Alhosin, Sherzad Rashid, Mélanie Burban, Cyril Auger, Valérie B. Schini-Kerth

**Affiliations:** CNRS UMR 7213 Laboratoire de Biophotonique et Pharmacologie, Université de Strasbourg, Faculté de Pharmacie, Illkirch, France; University of Illinois at Chicago, United States of America

## Abstract

**Aims:**

Omega-3 fatty acid products containing eicosapentaenoic acid (EPA) and docosahexaenoic acid (DHA) have vasoprotective effects, in part, by stimulating the endothelial formation of nitric oxide (NO). This study determined the role of the EPA:DHA ratio and amount, and characterized the mechanism leading to endothelial NO synthase (eNOS) activation.

**Methods and Results:**

EPA:DHA 6∶1 and 9∶1 caused significantly greater endothelium-dependent relaxations in porcine coronary artery rings than EPA:DHA 3∶1, 1∶1, 1∶3, 1∶6, 1∶9, EPA and DHA alone, and EPA:DHA 6∶1 with a reduced EPA + DHA amount, which were inhibited by an eNOS inhibitor. Relaxations to EPA:DHA 6∶1 were insensitive to cyclooxygenase inhibition, and reduced by inhibitors of either oxidative stress, Src kinase, PI3-kinase, p38 MAPK, MEK, or JNK. EPA:DHA 6∶1 induced phosphorylation of Src, Akt, p38 MAPK, ERK, JNK and eNOS; these effects were inhibited by MnTMPyP. EPA:DHA 6∶1 induced the endothelial formation of ROS in coronary artery sections as assessed by dihydroethidium, and of superoxide anions and hydrogen peroxide in cultured endothelial cells as assessed by electron spin resonance with the spin probe CMH, and the Amplex Red based assay, respectively.

**Conclusion:**

Omega-3 fatty acids cause endothelium-dependent NO-mediated relaxations in coronary artery rings, which are dependent on the EPA:DHA ratio and amount, and involve an intracellular activation of the redox-sensitive PI3-kinase/Akt and MAPKs pathways to activate eNOS.

## Introduction

Many prospective cohort studies and meta-analyses have provided evidence that fish consumption, especially oily fish (e.g., salmon, trout, herring, sardines, and mackerel), with high amounts of omega-3 fatty acids, including eicosapentaenoic acid (EPA, C20∶5 ω-3) and docosahexaenoic acid (DHA, C22∶6 ω-3), or supplementation with omega-3 fatty acids reduce cardiovascular mortality in patients with heart disease [1], [2]. In the GISSI-P trial published in 1999 for patients surviving a recent myocardial infarction, intake of 289 mg EPA plus 577 mg DHA per day reduced significantly fatal cardiovascular diseases by 30%, fatal coronary artery diseases by 35%, and sudden death by 45% [3]. However, more recent double-blind trials did not show an effect of an additional amount of EPA plus DHA on major cardiovascular endpoints in patients with coronary artery disease or after myocardial infarction [4], [5]. Such differences have been explained by differences in study design, the fact that patients in the more recent trials were optimally treated not only by antithrombotics but also by antihypertensives and statins, and possibly also due to the use of different doses, sources (oily fish or fish-oil supplements), and formulation of EPA and/or DHA [6]. The beneficial effect of omega-3 fatty acids on the cardiovascular system has been suggested to involve several mechanisms and in particular their ability to induce anti-inflammatory and anti-arrhythmic effects, to improve the lipid profile by lowering triglyceride levels, to prevent plaque development and possibly also to promote plaque stabilization, and to inhibit platelet aggregation [6]. In addition, the beneficial effect might also be due to their ability to improve endothelial dysfunction, which may represent a very early step in atherogenesis and is characterized by blunted endothelium-dependent vasodilatation mostly due to a reduced bioavailability of endothelium-derived nitric oxide (NO), and, often, endothelium-derived hyperpolarization (EDH). Indeed, chronic intake of fish oils increased endothelium-dependent relaxations in normal and in hypercholesterolemic and atherosclerotic porcine coronary arteries [7], [8]. An improved endothelial function as assessed by flow-mediated vasodilatation was observed in humans after intake of fish-derived products or omega-3 fatty acids [9], [10]. Moreover, intake of EPA and DHA improved the postprandial macro- and microvascular function in patients with type 2 diabetes mellitus [11]. EPA has been shown to cause endothelium-dependent relaxations of isolated sheep pulmonary arteries, and to increase endothelial NO synthase (eNOS) activity and NO formation in cultured endothelial cells [12]-[14]. In addition, EPA but not DHA has also been shown to cause cerebral microvascular vasodilatation involving arachidonic acid metabolites [15]. On the other hand, DHA but not EPA supplementation improved forearm microvascular reactivity in overweight hyperlipidemic men [16]. In order to better understand the omega-3 fatty acid-induced endothelial protection, the ability of different omega-3 fatty acid products (EPA, DHA, different ratios of EPA:DHA, different content of EPA and DHA) to cause endothelium-dependent relaxations in porcine coronary arteries was evaluated. Thereafter, the endothelium-dependent relaxation to one of the most active omega-3 products was further characterized and in particular the role of endothelium-derived NO, EDH and vasoactive prostanoids, and the signal transduction pathway leading to eNOS activation were determined.

## Materials and Methods

### Vascular reactivity studies

Pig hearts were collected from the local slaughterhouse (Copvial, Holtzheim) and vascular reactivity was assessed as indicated previously [17], [18]. Briefly, left circumflex coronary arteries were excised, cleaned of loose connective tissue and flushed with PBS without calcium to remove remaining blood. Rings of porcine coronary arteries (4-5 mm in length) were then suspended in organ baths containing oxygenated (95% O_2_; 5% CO_2_) Krebs bicarbonate solution (composition in mM: NaCl 119, KCl 4.7, KH_2_PO_4_ 1.18, MgSO_4_ 1.18, CaCl_2_ 1.25, NaHCO_3_ 25 and D-glucose 11, pH 7.4, 37°C) for the determination of changes in isometric tension (basal tension 5 g). The integrity of the endothelium was checked with bradykinin (0.3 µM). Rings were contracted (about 80% maximal contraction) with U46619, a thromboxane A2 receptor agonist, before construction of a concentration-relaxation curve to an omega-3 fatty acid product. In some experiments, rings were exposed to an inhibitor for 30 min before the addition of U46619.

### Culture of coronary artery endothelial cells

Endothelial cells from coronary arteries were isolated from porcine left circumflex coronary arteries by collagenase treatment (type I, Worthington, 1 mg/ml for 12 min at 37°C), and cultured in culture dishes containing medium MCDB 131 (Invitrogen, Saint Aubin, France) supplemented with 15% fetal calf serum, penicillin (100 U/ml), streptomycin (100 U/ml), fungizone (250 µg/ml), and L-glutamine (2 mM) (all from Cambrex, Wiesbaden, Germany), and grown for 48–72 h. Cells (first passage) were exposed to serum-free culture medium in the presence of 0.1% bovine serum albumin (Euromedex, Souffelweyersheim, France) for 6 h prior to treatment.

### Western blot analysis

Endothelial cells were lysed in extraction buffer (composition in mM: Tris/HCl 20 (Euromedex), NaCl 150, Na_3_VO_4_ 1, sodium pyrophosphate 10, NaF 20, okadaic acid 0.01, a tablet of protease inhibitor (Complete, Roche, Meylan, France) and 1% Triton X-100 (Euromedex); pH 7.5). Total proteins (10 µg) were separated on 10–12% SDS-polyacrylamide gels at 100 V for 2 h. Separated proteins were transferred electrophoretically onto polyvinylidine difluoride membranes (GE Healthcare, Velizy-Villacoublay, France) at 100 V for 120 min. Membranes were blocked with buffer containing 5% bovine serum albumin, Tris-buffered saline solution (Euromedex) and 0.1% Tween 20 (Sigma-Aldrich, Saint-Quentin Fallavier, France) for 1 h. Membranes were incubated with a primary antibody (p-Src Ser17, p-Akt Ser473, p-p38 MAPK Thr180/Tyr182, p-JNK Thr183/Tyr185, p-ERK Thr202/Tyr204, p-eNOS Ser1177 from rabbit; eNOS and β-tubulin from mouse, Cell Signaling Technology; dilution of 1∶1,000) overnight at 4°C. After washing, membranes were incubated with the appropriate horseradish peroxidase-conjugated secondary antibody (diluted to 1∶10,000 for anti-mouse antibody and 1∶5,000 for anti-rabbit antibody, Cell Signaling Technology, Danvers, MA, USA) at room temperature for 60 min. Immunoreactive bands were detected by enhanced chemiluminescence (GE Healthcare).

### Determination of the vascular formation of ROS

#### Dihydroethidium staining

Porcine coronary arteries (5–7 mm length) were embedded in OCT compound (Sakura Finetek, Villeneuve d'Ascq, France) and frozen in a nitrogen bath for cryostat sections. The redox-sensitive fluorescent dye dihydroethidium (DHE, 2.5 µM) was applied onto 25 µm unfixed cryosections of coronary arteries for 30 min at 37°C in a light-protected humidified chamber before the addition of EPA:DHA 6∶1 (0.4% v/v) for 30 min. In some experiments, sections were incubated with a pharmacological tool for 30 min at 37°C before the addition of DHE. Sections were then washed, mounted in fluorescence mounting medium (Dako, Les Ulis, France) and cover-slipped. Images were obtained using a Leica SP2 UV DM IRBE laser scanning confocal microscope (Leica Microsystem, Nanterre, France). Quantification of staining levels was performed using FIJI GPL v2 software.

#### Electron spin resonance (ESR)

ESR was used to determine whether EPA:DHA 6∶1 stimulates the endothelial formation of superoxide anions in cultured endothelial cells using the superoxide anion cell-permeable spin probe CMH following the method described [19].

Endothelial cells were incubated with either solvent, SOD or PEG-SOD followed by treatment with EPA:DHA 6∶1 (0.4% v/v) in Krebs-HEPES solution containing 500 µM of 1-hydroxy-3-methoxycarbonyl-2,2,5,5-tetramethylpyrrolidin (CMH, Noxygen, Germany), deferoxamine (25 µM) and DETC (5 µM) under constant temperature (37°C) for 30 min. The reaction was stopped by putting the samples on ice, subsequently they were frozen in liquid N_2_ and analyzed by ESR at 77 K in a Dewar flask using a MS100 spectrometer (Magnettech Ltd., Berlin, Germany). Instrument settings were: temperature 77 K, microwave frequency 9.34 GHz, microwave power 10 mW, amplitude of modulation 100 mT, modulation of frequency 100 kHz, and 60 s of sweep time. Superoxide anions cannot be directly measured by ESR due to their short half-life. Therefore, the CMH spin-trapping reagent has been used. It reacts with superoxide anions to generate the stable CM^•^ radical, which gives a characteristic triplet signal ESR spectra, with an amplitude directly proportional to the quantity of CM^•^ present in the sample. The quantification of the signal is based on the mean of the height (amplitude) of the three signals. Values are expressed in signal amplitude (Amplitude, arbitrary units).

#### Amplex Red assay

Hydrogen peroxide measurements in cultured endothelial cells were made using the horseradish peroxidase-linked Amplex Red fluorescence assay kit as recommended (Invitrogen, Paisley, UK). Endothelial cells were incubated with either solvent, catalase or PEG-catalase for 30 min and then they were treated with EPA:DHA 6∶1 (0.4% v/v) for 45 min. Amplex Red (50 µM) and horseradish peroxidase type II (0.1 U/ml) were added to the supernatant samples (100 µl). Fluorescence readings were made in triplicate in a 96-well plate, and Ex/Em  = 530/580 nm.

### Chemicals

All products were from Sigma-Aldrich (Saint-Quentin Fallavier, France), except wortmannin, PP2 and MnTMPyP from Enzo Life Sciences (Lausen, Switzerland). Omega-3 fatty acid products (Table 1) were provided by Pivotal Therapeutics, Inc (Woodbridge, ON, Canada).

**Table 1:** Characteristics of the different omega-3 fatty acid products.

Fatty acids	Purity *(in %)*	Ratio EPA:DHA	Content of Omega-3 EPA-DHA as EE		Sum Omega-3 as EE (mg/g)
			EPA (mg/g)	DHA (mg/g)	
EPA:DHA	93.4	1∶1	476	386	934
EPA:DHA	91.3	6∶1	694	121	913
EPA:DHA	46.0	6∶1	352	65	460
EPA	99.5		991		995
DHA	98.6			945	986

Fatty acids contents are given in mg/g expressed as ethyl ester (EE).

### Statistical analysis

All values are expressed as means ± SEM of n different experiments. Statistical analysis was performed using Student's paired *t* test or a two-way analysis of variance test followed by Bonferroni's post-hoc test as appropriate using Graphpad Prism software (version 5.04 for Windows, GraphPad Software, Inc., CA, USA). *P*<0.05 was considered to be statistically significant.

## Results

### The omega-3 fatty acid-induced endothelium-dependent relaxations are dependent on the EPA:DHA ratio and content

**Figure 1:**
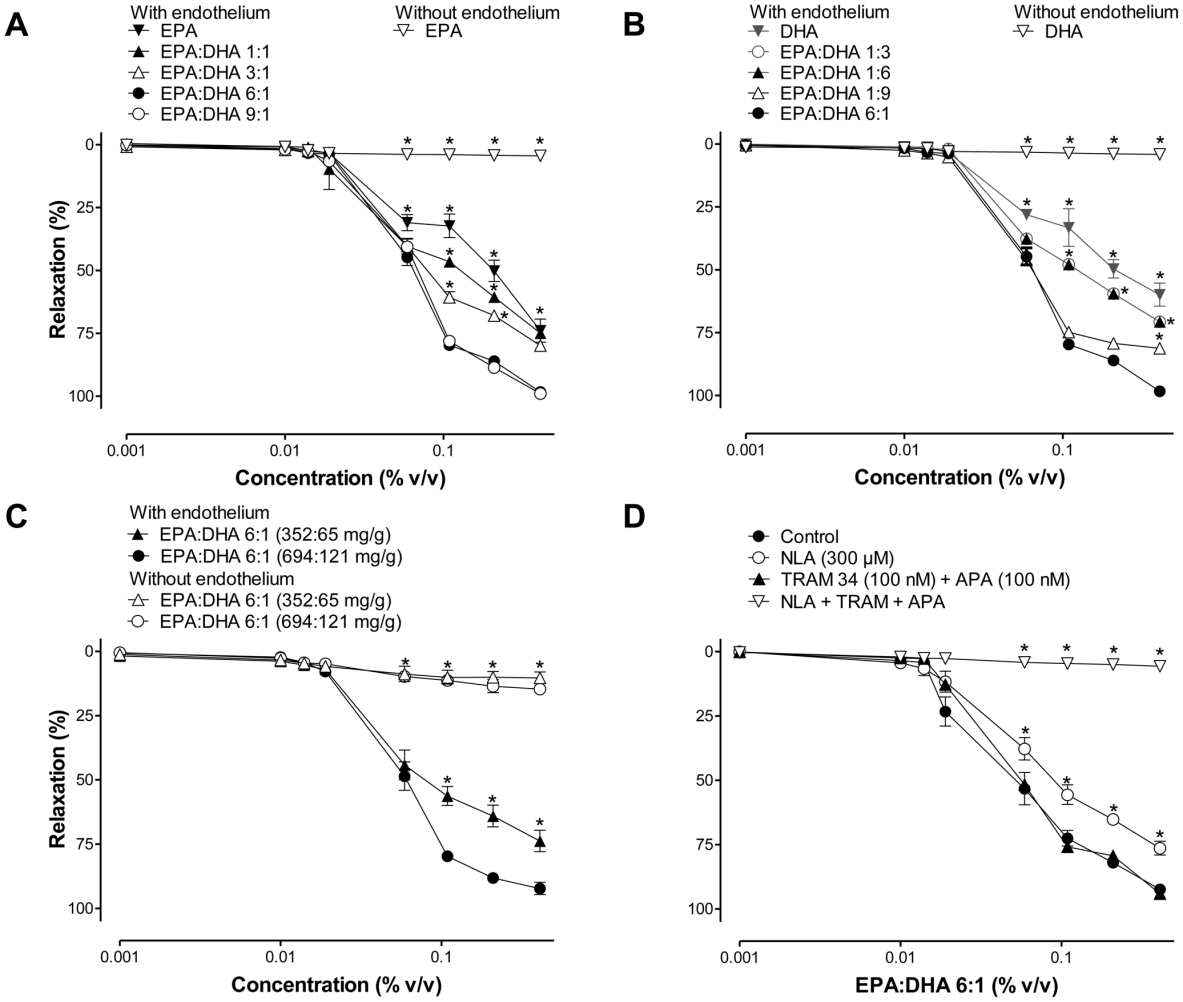
Omega-3 fatty acids cause endothelium-dependent relaxations, which are dependent on the EPA:DHA ratio and the EPA plus DHA content. (A, B, C) Coronary artery rings with endothelium were contracted with U46619 before the addition of increasing concentrations of an omega-3 fatty acid product. (D) Rings were incubated with either L-NA (NO synthase inhibitor), TRAM34 plus apamin (inhibitors of endothelium-dependent hyperpolarization) or the three inhibitors together for 30 min before the contraction to U46619 and the subsequent relaxation to EPA:DHA 6∶1. All experiments were performed in the presence of indomethacin (10 µM) to prevent the formation of vasoactive prostanoids. Results are expressed as means ± SEM of 5 different experiments. *P<0.05 versus (A, B) EPA:DHA 6∶1 with endothelium, (C) EPA:DHA 6∶1 (694∶121 mg/g) with endothelium, and (D) control.

To study the vasoactive effect of omega-3 fatty acid products, coronary artery rings with endothelium were contracted with U46619 before the addition of increasing concentrations of an omega-3 fatty acid product. Both EPA and DHA, the two major omega-3 fatty acid products, induced concentration-dependent relaxations in coronary artery rings with endothelium whereas no such effect was observed in those without endothelium (Figures 1A and B). EPA and DHA induced similar relaxations except at 0.4% (v/v) where relaxations to EPA were slightly but significantly greater than those to DHA (77.7±10.3 and 64.6±12.8%, respectively, Figures 1A and B). Next, the ability of different EPA:DHA ratios to induce endothelium-dependent relaxations was determined. EPA:DHA with different ratios induced concentration-dependent relaxations in intact coronary artery rings (Figures 1A and B). However, relaxations to EPA:DHA 6∶1 and 9∶1 were significantly greater than those to EPA:DHA 3∶1, 1∶1, 1∶3, 1∶6, and 1∶9 (Figures 1A and B). An increased relaxation was also observed in response to an EPA:DHA 6∶1 product having a high EPA plus DHA content (694∶121 mg/g) compared to one having a low content (352∶65 mg/g, Figure 1C). Altogether, these findings indicate that omega-3 fatty acids are inducers of endothelium-dependent relaxations in coronary artery rings, and that this effect is dependent on the EPA:DHA ratio and on the EPA plus DHA amount.

### EPA:DHA 6∶1 induces endothelium-dependent relaxations involving predominantly NO and also, to some extent, endothelium-dependent hyperpolarization

Next, experiments were performed to determine the role of the different endothelium-derived relaxing factors in the EPA:DHA 6∶1-induced relaxation of the coronary artery. Relaxations to EPA:DHA 6∶1 were slightly but significantly inhibited by L-NA (a competitive inhibitor of eNOS), not affected by inhibitors of endothelium-dependent hyperpolarization-mediated responses, TRAM 34 plus apamin (inhibitors of Ca^2+^-dependent potassium channels of intermediate and low conductance IK_Ca_ and SK_Ca_, respectively), and abolished by the combination of L-NA plus TRAM 34 and apamin (Figure 1D). In addition, they were not affected by indomethacin (cyclooxygenase inhibitor; relaxations at 0.4% v/v were 94.8 ± 3.2% and 95.8 ± 3.2% in the absence and presence of indomethacin, n = 5). These findings indicate that EPA:DHA 6∶1 induces endothelium-dependent relaxations in coronary artery rings involving predominantly NO, and also, to some extent, EDH but not vasoactive prostanoids.

### EPA:DHA 6∶1 induces redox-sensitive NO-mediated relaxations involving Src kinase, PI3-kinase, and MAPKs

**Figure 2:**
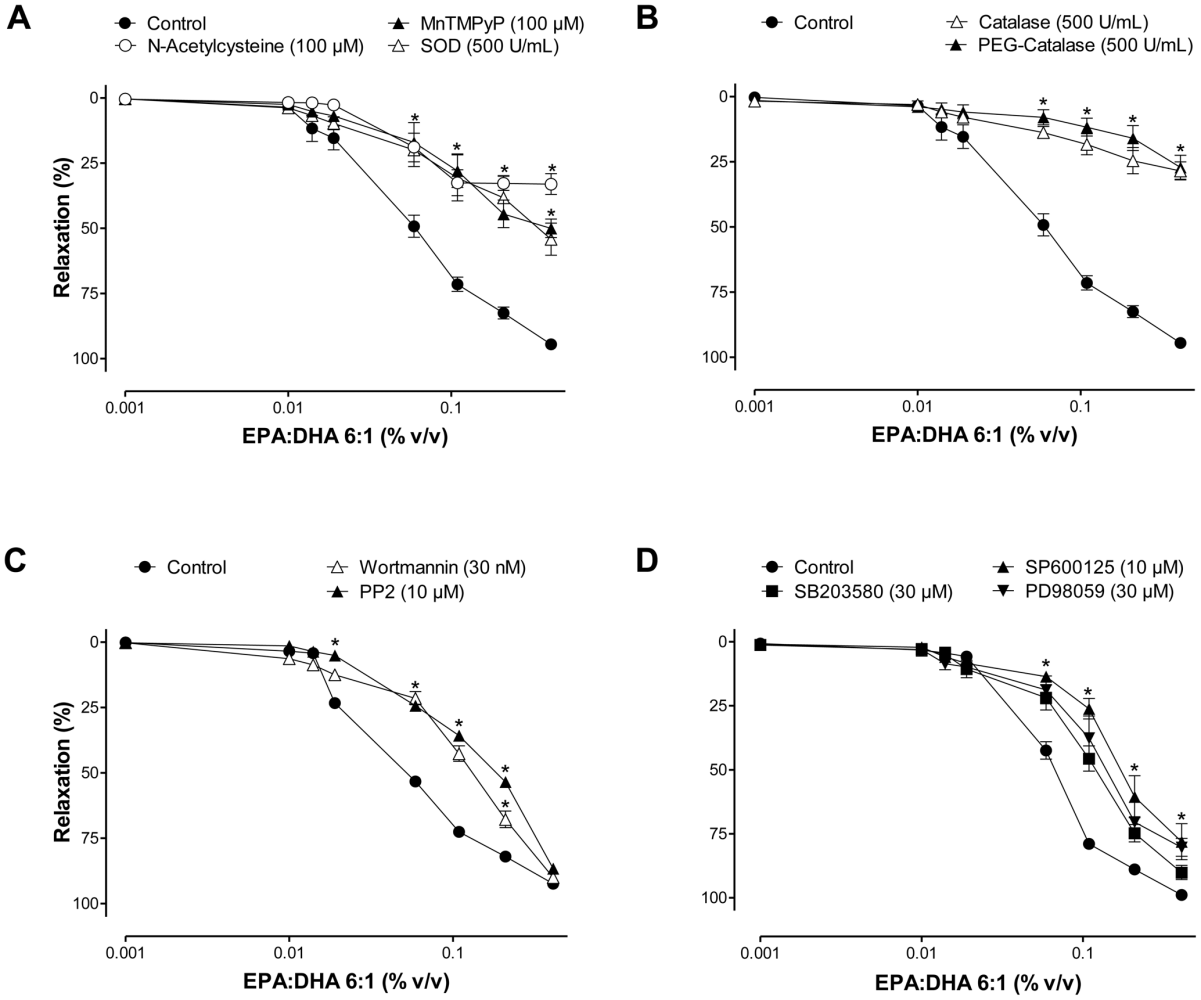
The EPA:DHA 6∶1-induced endothelium-dependent NO-mediated relaxation involves a redox-sensitive event. Coronary artery rings with endothelium were incubated either with (A) an antioxidant (N-acetylcysteine), an extracellular scavenger of superoxide anions (SOD), a membrane permeant mimetic of superoxide dismutase (MnTMPyP), (B) extracellular and membrane permeant analogue of catalase (catalase and PEG-catalase, respectively), (C) an inhibitor of either Src kinase (PP2) or PI3-kinase (wortmannin), (D) an inhibitor of either p38 MAPK (SB203580), MEK (PD98059) or JNK (SP600125) for 30 min before the addition of U46619 and the subsequent relaxation to EPA:DHA 6∶1. All experiments were performed in the presence of indomethacin (10 µM) and TRAM34 plus apamin (both at 100 nM) to rule out the formation of vasoactive prostanoids and endothelium-dependent hyperpolarization-mediated responses, respectively. Results are presented as means ± SEM of 5 different experiments. *P<0.05 versus control.

Besides the calcium-calmodulin complex, the redox-sensitive Src/PI3-kinase/Akt and MAPKs pathways have also been shown to activate eNOS in endothelial cells in response to both physiological activators such as shear stress and estrogens, and natural products such as grape-derived polyphenols and tea catechins [18], [20]-[23]. Since the EPA-induced activation of eNOS is a calcium-independent event [12], experiments were performed to determine the role of redox-sensitive Src/PI3-kinase/Akt and MAPKs pathways in the NO-mediated relaxations to EPA:DHA 6∶1. For this purpose, all subsequent experiments were performed in the presence of indomethacin and TRAM34 plus apamin to rule out the formation of vasoactive prostanoids and EDH-mediated responses, respectively. Relaxations to EPA:DHA 6∶1 were significantly reduced by the antioxidant N-acetylcysteine, the membrane permeant mimetic of SOD and the membrane permeant catalase (MnTMPyP and PEG-catalase, respectively) and native SOD and catalase (Figures 2 A and B). In addition, they were also reduced by PP2 (a Src kinase inhibitor), wortmannin (a PI3-kinase inhibitor), SB203580 (a p38 MAPK inhibitor), SP600125 (a JNK inhibitor), PD98059 (a MEK inhibitor, Figures 2C and D). In contrast to EPA:DHA 6∶1, relaxations to bradykinin were not affected by either PD98059, SP600125, MnTMPyP, PP2 or wortmannin (data not shown) [24]-[26]. Thus, these findings indicate that the EPA:DHA 6∶1-induced NO-mediated relaxation is critically dependent on a redox-sensitive event involving Src kinase/PI3-kinase and MAPKs pathways.

### EPA:DHA 6∶1 induces the redox-sensitive Src/PI3-kinase/Akt- and MAPKs-dependent phosphorylation of eNOS at Ser 1177

**Figure 3:**
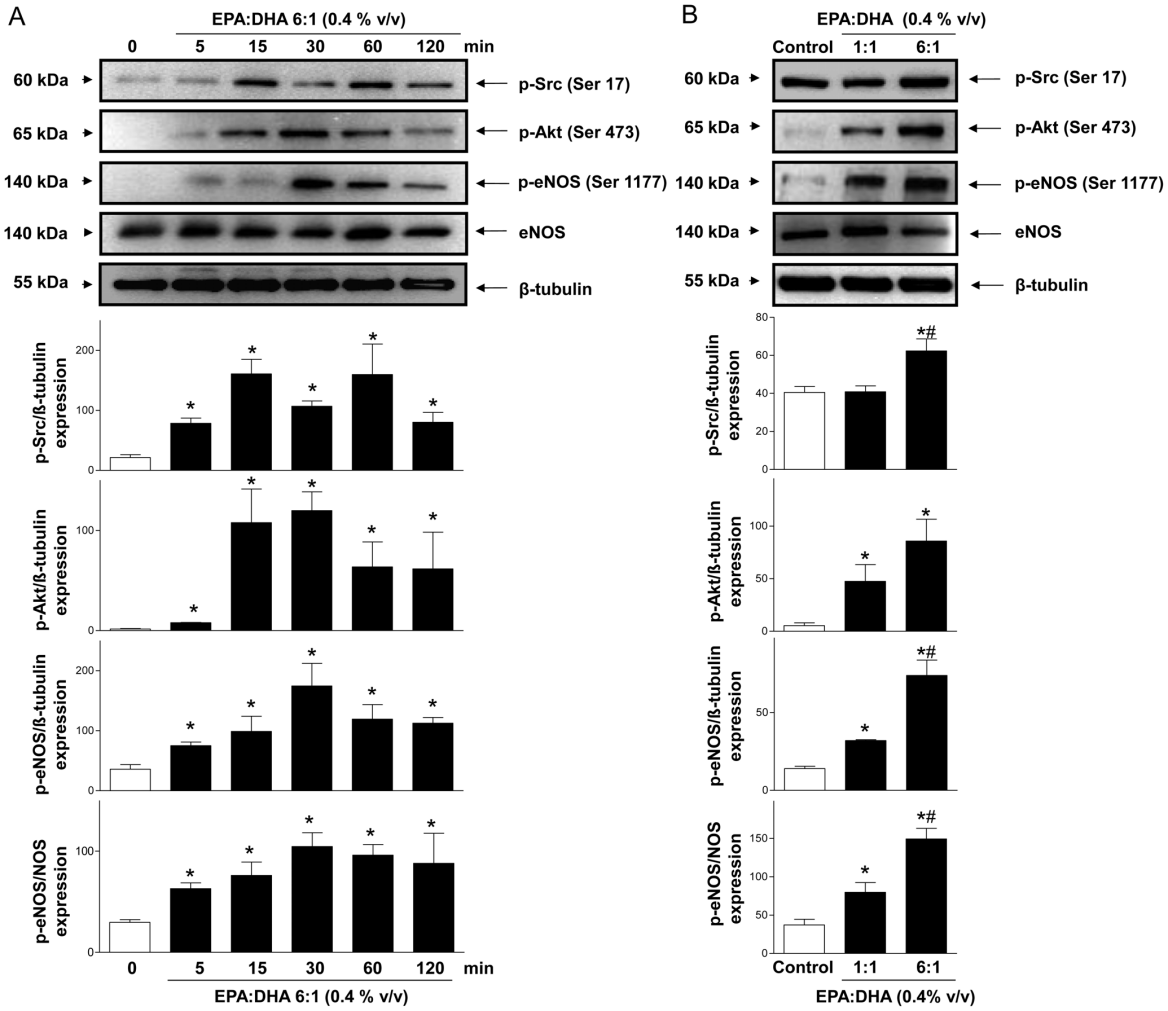
EPA:DHA 6∶1 induces the phosphorylation of Src, Akt and eNOS in porcine coronary artery endothelial cells in a time- and EPA:DHA ratio-dependent manner. (A) Cultured endothelial cells were exposed to EPA:DHA 6∶1 (0.4% v/v) for the indicated times at 37°C. (B) Cultured endothelial cells were exposed to products with either an EPA:DHA ratio of 6∶1 or 1∶1 at the concentration of 0.4% (v/v) for 30 min at 37°C. Representative immunoblots of p-Src, p-Akt and p-eNOS proteins as assessed by Western blot analysis (top), and corresponding cumulative data (bottom). Results are shown as means ± SEM of 3 different experiments. *P<0.05 versus control,^ #^ *P*<0.05 versus EPA:DHA 6∶1 treatment.

**Figure 4:**
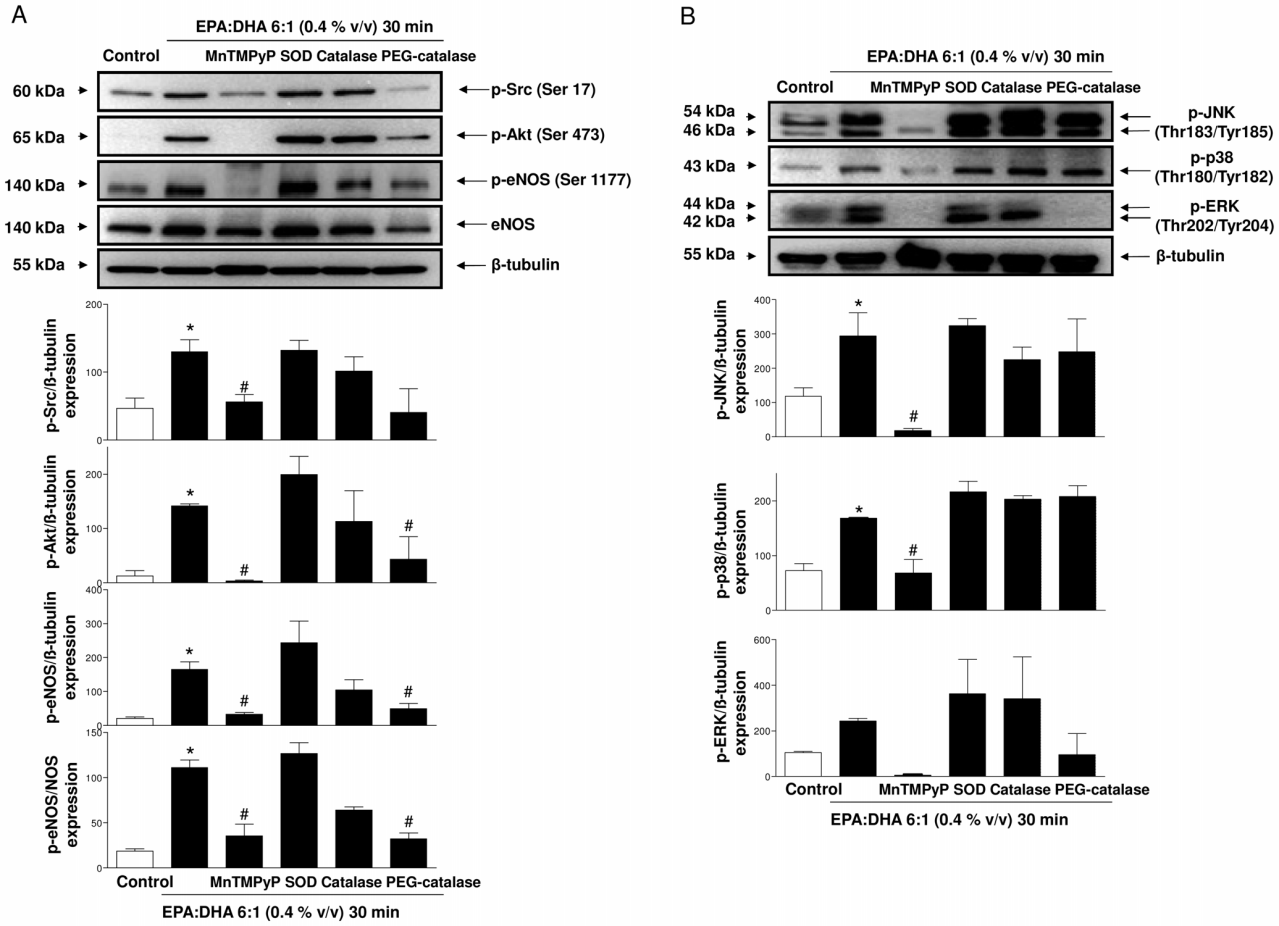
The EPA:DHA 6∶1-induced phosphorylation of Src, Akt, eNOS, JNK, p38 MAPK and ERK is a redox-sensitive event involving predominantly superoxide anions in endothelial cells. Endothelial cells were incubated either with solvent, MnTMPyP (100 µM), SOD (500 U/ml), catalase (500 U/ml) or PEG-catalase (500 U/ml) for 30 min before the addition of EPA:DHA 6∶1 (0.4% v/v) for 30 min. Representative immunoblots for detection of (A) p-Src, p-Akt and p-eNOS and (B) p-JNK, p-p38 MAPK and p-ERK (top), and corresponding cumulative data (bottom). Results are shown as means ± SEM of 3-4 different experiments except for p-ERK (n = 2). *P<0.05 versus control,^ #^ *P*<0.05 versus EPA:DHA 6∶1 treatment.

To obtain further evidence that EPA:DHA 6∶1 activates Src, Akt, MAPKs and eNOS, endothelial cells were cultured from coronary arteries and the phosphorylation level of these proteins at activator sites (Ser17 for p-Src, Ser473 for p-Akt, Thr183/Tyr185 for p-JNK, Thr180/Tyr182 for p-p38 MAPK, Thr202/Tyr204 for p-ERK, and Ser1177 for p-eNOS) was examined by Western blot analysis. EPA:DHA 6∶1 at 0.4% v/v caused a time-dependent phosphorylation of Src, Akt and eNOS with significantly increased levels observed within 5 min and, thereafter, these signals remained elevated at least up to 2 h (Figure 3A). In addition, EPA:DHA 6∶1 increased to a greater extent the phosphorylation of Src, Akt and eNOS than EPA:DHA 1∶1 (Figure 3B). EPA:DHA 6∶1 (0.4% v/v) also significantly increased the phosphorylation level of p38 MAPK, JNK and ERK (Figure 4B). The possibility that the redox-sensitive event triggers the Src/PI3-kinase/Akt and MAPKs pathways leading to eNOS phosphorylation was examined. The EPA:DHA 6∶1-induced phosphorylation of Src, Akt and eNOS was markedly reduced by MnTMPyP and PEG-catalase and not significantly affected by native SOD and catalase (Figure 4A). The EPA:DHA 6∶1-induced phosphorylation of JNK, p38 MAPK and ERK was also markedly reduced by MnTMPyP whereas SOD, catalase had only minor effects (Figure 4B). PEG-catalase did not significantly affect the phosphorylation of JNK and p38 MAPK but reduced that of ERK (Figure 4B). Thus, these findings suggest that intracellular ROS, in particular superoxide anions, are upstream activators of the Src/PI3-kinase/Akt and MAPKs pathways leading to eNOS activation in response to EPA:DHA 6∶1.

### EPA:DHA 6∶1 induces a pro-oxidant response in coronary artery endothelial cells

**Figure 5:**
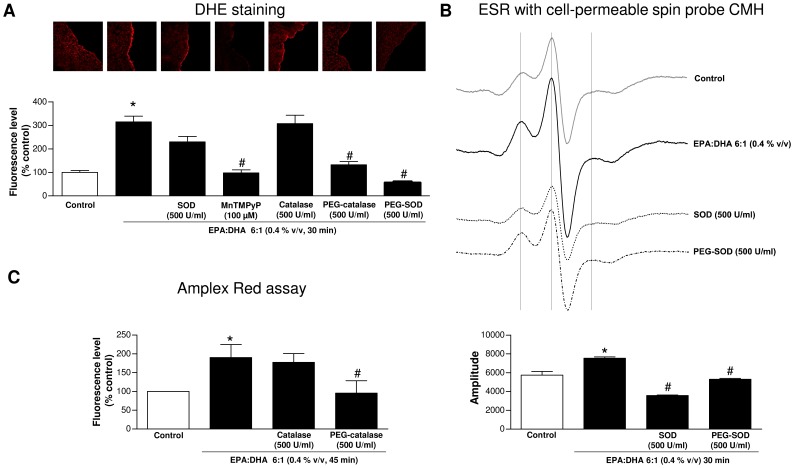
EPA:DHA 6∶1 induces the formation of ROS involving superoxide anions and hydrogen peroxide in endothelial cells. A) The EPA:DHA 6∶1-induced oxidative stress was assessed in coronary artery sections with endothelium using the redox-sensitive probe dihydroethidium (2.5 µM for 30 min). The EPA:DHA 6∶1-induced formation of B) superoxide anions in cultured endothelial cells was assessed using electron spin resonance and the cell permeable spin probe CMH, and C) of hydrogen peroxide using the Amplex Red assay. B) Top: Representative original traces showing the three signals used for quantification (vertical bars). Bottom: Corresponding cumulative data. Means of the amplitude (height) of the three signals was measured for each spectrum. Results are shown as means ± SEM of 3-5 different experiments. *P<0.05 versus control,^ #^ *P*<0.05 versus EPA: DHA 6∶1 treatment.

Experiments were performed to determine whether EPA:DHA 6∶1 stimulates the endothelial formation of ROS in coronary artery sections using the redox-sensitive fluorescent probe dihydroethidium (DHE). EPA:DHA 6∶1 markedly increased the fluorescence signal in the endothelium whereas the signal in the smooth muscle was minimally affected (Figure 5A). The stimulatory effect of EPA:DHA 6∶1 was markedly reduced by MnTMPyP and PEG-SOD, and, to a lesser extent, by PEG-catalase, and not affected by SOD and catalase (Figure 5A). In addition, EPA:DHA 6∶1 significantly increased the formation of superoxide anions in cultured endothelial cells as assessed using ESR and the cell permeable spin probe CMH, and this effect was inhibited by SOD and PEG-SOD (Figure 5B). Moreover, EPA:DHA 6∶1 significantly increased the formation of hydrogen peroxide in cultured endothelial cells as assessed using the Amplex Red based assay, and this effect was inhibited by PEG-catalase but not by catalase (Figure 5C). Altogether, these findings indicate that EPA:DHA 6∶1 induces a pro-oxidant response in endothelial cells involving both superoxide anions and hydrogen peroxide.

## Discussion

The major findings of the present study indicate that the ability of omega-3 fatty acid products to cause endothelium-dependent relaxations involving predominantly NO and also, to some extent endothelium-dependent hyperpolarization in coronary arteries, depends on the formulation and the content of EPA and DHA, and that the EPA:DHA 6∶1 is a superior formulation than EPA:DHA 1∶1. They further indicate that the stimulatory effect of EPA:DHA 6∶1 leading to eNOS activation involves the Src/PI3-kinase/Akt and MAPKs pathways with the subsequent phosphorylation of eNOS at the activator site Ser1177. Moreover, a redox-sensitive intracellular event involving superoxide anions and hydrogen peroxide has been identified as an early event triggering the activation of the Src/PI3-kinase/Akt and MAPKs pathways in endothelial cells. Thus, endothelial cells appear to act as special sensors of omega-3 fatty acids in the arterial wall to increase in a sustained manner the eNOS-derived formation of NO, and, hence, vascular protection.

The evaluation of the ability of different omega-3 fatty acid products to relax porcine coronary arteries has indicated that EPA, DHA and formulations with different ratios of EPA:DHA induced pronounced relaxations in intact arterial preparations whereas they had little effects in rings without endothelium. Moreover, although EPA and DHA alone evoked similar concentration-relaxation curves up to 0.2% (v/v), their combination in the formulation EPA:DHA 6∶1 and 9∶1 demonstrated superiority to the formulation EPA:DHA 3∶1, 1∶1, 1∶3, 1∶6 and 1∶9. The mechanism accounting for such a synergistic effect remains unclear. Potential explanations could be that the integration of omega-3 fatty acids into membrane lipids and/or that the stability of the omega-3 fatty acids are dependent on an optimal EPA:DHA ratio. Indeed, EPA but not DHA has also been shown to cause cerebral microvascular vasodilatation involving arachidonic acid metabolites [15], whereas DHA but not EPA supplementation improved forearm microvascular reactivity in overweight hyperlipidemic men [16]. The characterization of the omega-3 fatty acid-induced endothelium-dependent relaxation has indicated a predominant role of NO, and also, to some extent, endothelium-dependent hyperpolarization whereas vasoactive prostanoids are not involved. Previous studies have also shown an increased endothelium-dependent relaxation or vasodilatation in response to the acute addition of EPA [12], and the chronic administration of EPA (3.5 g daily) and DHA (1.5 g daily) in pigs [7], [27], and an increased vasodilatation to EPA (1.8 g daily) in humans [28].

Although omega-3 fatty acids are potent inducers of the endothelial formation of NO, the underlying mechanism remains poorly characterized. Activation of eNOS can be triggered by elevations in intracellular calcium by calcium elevating agonists such as bradykinin and thrombin, subsequently to the binding of the calcium-calmodulin complex to eNOS and the dissociation of the enzyme from its inhibitor caveolin [29]. Although EPA induced elevations in intracellular calcium in endothelial cells, these responses were very small and did not contribute to eNOS activation [12]. EPA and DHA have also been shown to alter caveolae microenvironment not only by modifying membrane lipid composition but also by changing distribution of major structural proteins, which may eventually lead to eNOS activation [30], [31]. The present findings provide evidence for an additional mechanism of eNOS activation by omega-3 fatty acids via the Src/PI3-kinase/Akt and MAPKs pathways leading to the phosphorylation of eNOS at the activator site Ser1177. Indeed, inhibitors of either Src kinase, PI3-kinase, JNK, p38 MAPK or MEK reduced NO-mediated relaxations to EPA:DHA 6∶1, and EPA:DHA 6∶1 increased the phosphorylation level of activator sites of Src, Akt, JNK, p38 MAPK, ERK and eNOS in endothelial cells. Of interest, the Src/PI3-kinase/Akt pathway has also been shown to mediate the stimulatory effect of other natural products containing high levels of polyphenols such as grape-derived products and tea catechins on eNOS [17], [18], [22], [23]. Similarly to polyphenols [17], [18], [22], [23], the intracellular formation of ROS, in particular superoxide anions and hydrogen peroxide, mediates the stimulatory effect on the Src/PI3-kinase/Akt and MAPKs pathways leading to eNOS activation in response to EPA:DHA 6∶1. Indeed, inhibitors of intracellular oxidative stress and in particular the membrane permeant mimetic of SOD, MnTMPyP, markedly reduced the relaxation and the phosphorylation of Src, Akt, JNK, p38 MAPK, ERK and eNOS in response to EPA:DHA 6∶1. Moreover, the redox-sensitive probe dihydroethidium provided direct evidence that EPA:DHA 6∶1 induced the formation of ROS in the native endothelium whereas the signal in the smooth muscle was minimally affected. Furthermore, EPA:DHA 6∶1-induced the generation of superoxide anions and hydrogen peroxide in cultured endothelial cells as assessed using the ESR technique with the cell permeable spin probe CMH and the Amplex Red assay, respectively. Native SOD and catalase did not affect the EPA:DHA 6∶1-induced phosphorylation of target proteins and the increased DHE fluorescence signal in the native endothelium of coronary artery sections. However, both compounds reduced NO-mediated relaxations, such a difference is most likely due to different experimental conditions and in particular to the high partial oxygen pressure in the vascular reactivity studies (aerated with 95% O_2_) compared to that in experiments using cultured endothelial cells (aerated with 18-20% O_2_) and coronary artery sections (ambient air, 21% O_2_). Besides endothelial cells, the induction of oxidative stress in response to omega-3 fatty acids has also been observed in several types of cancer cells including MCF-7 [32], and vascular smooth muscle cells to induce apoptosis [33]. The oxidative stress-induced apoptosis has been suggested to involve lipid peroxidation [32], and a calcium-regulated event [33].

In conclusion, the ability of omega-3 fatty acids to stimulate the endothelial formation of NO is dependent on the ratio and amount of the EPA:DHA formulation, and it involves an original pathway triggered by the intracellular oxidative stress leading to the activation of the Src/PI3-kinase/Akt and MAPKs pathways to increase ultimately eNOS activity.
